# Sex Differences in Mitochondrial Function: Endocrine Regulation, Immunometabolic Signaling, and Implications for Health and Disease

**DOI:** 10.3390/ijms27114966

**Published:** 2026-05-30

**Authors:** Hanna Bynum, Kristin S. Edwards

**Affiliations:** Department of Pharmacology and Toxicology, Women’s Health Research Center, Mississippi Center of Excellence in Perinatal Research, University of Mississippi Medical Center, Jackson, MS 39216, USA; hbynum1@umc.edu

**Keywords:** mitochondria, sex differences, sex hormones, estrogen, testosterone, oxidative phosphorylation, mitochondrial dynamics, mitochondrial reactive oxygen species, endocrine disorders, immunometabolism

## Abstract

Mitochondria are central regulators of cellular bioenergetics, redox balance, and signaling pathways that integrate metabolic and immune responses. Emerging evidence indicates that biological sex is an important determinant of mitochondrial function, in part through the regulatory effects of sex hormones on mitochondrial biogenesis, oxidative phosphorylation, reactive oxygen species production, and quality control mechanisms. Estrogen, testosterone, and progesterone differentially modulate mitochondrial dynamics, substrate utilization, antioxidant capacity, and immune signaling, resulting in distinct mitochondrial phenotypes that may influence disease susceptibility across the lifespan. In this review, we synthesize current knowledge on the mechanistic basis of sex differences in mitochondrial function and highlight mitochondria as key mediators linking endocrine signaling to immunometabolic regulation. We discuss how mitochondrial-derived signals, including mitochondrial reactive oxygen species, mitochondrial DNA release, and cardiolipin exposure, activate inflammatory pathways such as NF-κB, cGAS–STING, and NLRP3 inflammasome signaling. These pathways may contribute to chronic inflammation, gut barrier dysfunction, and systemic metabolic disruption. We further examine the impact of major endocrine transitions, including pregnancy, the postpartum period, menopause, and androgen imbalance in conditions such as polycystic ovary syndrome, on mitochondrial function and disease risk. Particular emphasis is placed on the gastrointestinal tract as a metabolically active and mitochondria-dependent interface, where mitochondrial dysfunction may contribute to epithelial barrier disruption, microbial dysbiosis, and systemic inflammation. Finally, we discuss emerging therapeutic strategies targeting mitochondrial function, including exercise, hormone-based therapies, mitochondria-targeted antioxidants, and interventions aimed at improving mitochondrial quality control. Understanding sex-specific mitochondrial regulation may provide a framework for improved endocrine stratification, mitochondrial phenotyping, and precision medicine approaches across diverse clinical contexts.

## 1. Introduction

Sex is a core biological variable shaping physiology and disease susceptibility across organ systems. Sex differences are consistently observed in cardiometabolic disease, neurodegeneration, immune-mediated disorders, and therapeutic responses, yet the molecular mechanisms that integrate endocrine signaling with cellular bioenergetics remain incompletely defined [1,2,3]. Mitochondria have emerged as central determinants of sex-associated phenotypes, functioning not only as ATP-producing organelles but also as signaling hubs that coordinate oxidative metabolism, redox signaling, calcium homeostasis, innate immune activation, and programmed cell death [4,5,6,7,8,9].

**Figure 1:**
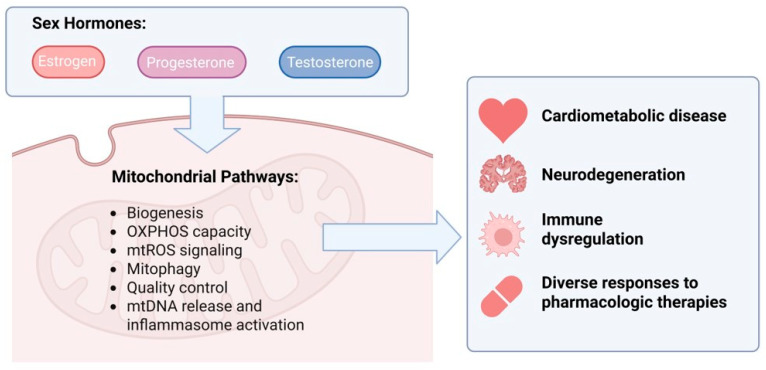
Sex hormone–mitochondria–disease axis. Sex hormones, including estrogens, androgens, and progesterone, regulate mitochondrial structure and function through coordinated effects on biogenesis, oxidative phosphorylation (OXPHOS), mitochondrial reactive oxygen species (mtROS) production, and mitochondrial quality control [10,11,12,13,14,15,16,17,18,19,20,21,22,23,24,25,26,27,28]. Estrogen signaling promotes mitochondrial biogenesis via PGC-1α–NRF1/2–TFAM pathways, enhances respiratory efficiency, and increases antioxidant capacity [10,11,12,13,14,15,16,29,30,31,32,33]. Androgen signaling modulates substrate utilization, lipid metabolism, and electron transport chain activity, whereas progesterone exerts context-dependent effects on mitochondrial respiration and immune metabolism [17,18,19,20,21,22,23,24,25,26,27,28]. These hormone-dependent mitochondrial adaptations may influence downstream disease outcomes, including cardiometabolic disease, neurodegeneration, immune dysregulation, reproductive dysfunction, and frailty [34,35,36,37,38,39,40]. This figure illustrates mitochondria as central integrators of endocrine signaling and disease susceptibility.

Mitochondria are increasingly recognized as endocrine-responsive signaling organelles that may contribute to sex differences in disease vulnerability. Because most mitochondrial proteins are nuclear encoded, hormonal regulation of transcriptional networks can shape mitochondrial content, intrinsic respiratory efficiency, redox buffering capacity, and quality control mechanisms. Estrogens, androgens, and progesterone regulate mitochondrial biogenesis, substrate selection, supercomplex stability, antioxidant defense, and mitophagy through coordinated genomic and non-genomic mechanisms [10,11,12,13,14,15,16]. Consequently, endocrine life-stage transitions and pathologic hormone imbalances provide clinically relevant models of mitochondrial remodeling. This integrative framework is illustrated in Figure 1.

While prior reviews have described sex differences in mitochondrial biology, few have integrated endocrine transitions, clinical hypogonadal states, and immune–metabolic signaling into a unified mechanistic framework. This review synthesizes these domains to position mitochondria as central mediators of sex-biased disease trajectories.

Importantly, mitochondrial content and mitochondrial functional capacity are not interchangeable measurements. Increases in mitochondrial DNA copy number, electron transport chain protein abundance, or mitochondrial mass do not necessarily reflect preserved respiratory efficiency or oxidative phosphorylation capacity. Throughout this review, the distinction between quantitative mitochondrial remodeling and qualitative mitochondrial function is emphasized to avoid overinterpretation of indirect mitochondrial endpoints.

Direct assessment of mitochondrial function requires functional bioenergetic measurements such as high-resolution respirometry, respiratory control ratio analysis, substrate-specific flux measurements, ATP production assays, and mitochondrial membrane potential assessment rather than reliance solely on static markers of mitochondrial abundance. Because mitochondrial responses to sex hormones are highly tissue-dependent and influenced by metabolic context, caution is required when extrapolating findings across organ systems or endocrine states. Understanding these distinctions is essential for interpreting how sex hormones influence mitochondrial biology across tissues, physiologic states, and disease conditions.

## 2. Background: Mitochondrial Biology and Core Functions

Mitochondria generate ATP through oxidative phosphorylation, coupling electron transfer across respiratory complexes to proton gradient formation and ATP synthesis at complex V [4,5]. Respiratory efficiency depends not only on the abundance of electron transport chain complexes but also on supercomplex assembly, cristae architecture, and inner mitochondrial membrane organization. These structural features are influenced by cardiolipin composition and OPA1-mediated cristae remodeling and can affect respiratory control ratio, proton leak, and intrinsic electron transport efficiency [41,42,43].

Beyond ATP production, mitochondria regulate central carbon metabolism through the tricarboxylic acid cycle, fatty acid β-oxidation, and pyruvate oxidation. Substrate selection is influenced by carnitine palmitoyltransferase-dependent fatty acid transport, pyruvate dehydrogenase activity, and tissue-specific nutrient availability [44,45,46]. Calcium exchange between the endoplasmic reticulum and mitochondria regulates ATP production, redox signaling, mitochondrial permeability transition, and apoptotic susceptibility, highlighting the importance of inter-organelle communication in mitochondrial homeostasis [7,47]. Mitochondrial outer membrane permeabilization further links mitochondrial stress to programmed cell death through cytochrome c release and downstream caspase activation [6].

Mitochondrial reactive oxygen species (mtROS) are generated from multiple mitochondrial sites, including electron transport chain complexes I and III as well as matrix-associated enzymes such as pyruvate dehydrogenase, α-ketoglutarate dehydrogenase, and dihydroorotate dehydrogenase. This broader view highlights the complexity of mitochondrial redox signaling beyond the electron transport chain alone [48,49,50,51,52]. Physiologic mtROS production supports adaptive redox-sensitive signaling pathways, including NRF2 activation and NF-κB-mediated transcription, whereas excessive mtROS can promote oxidative damage, inflammatory amplification, and impaired mitochondrial quality control.

**Figure 2:**
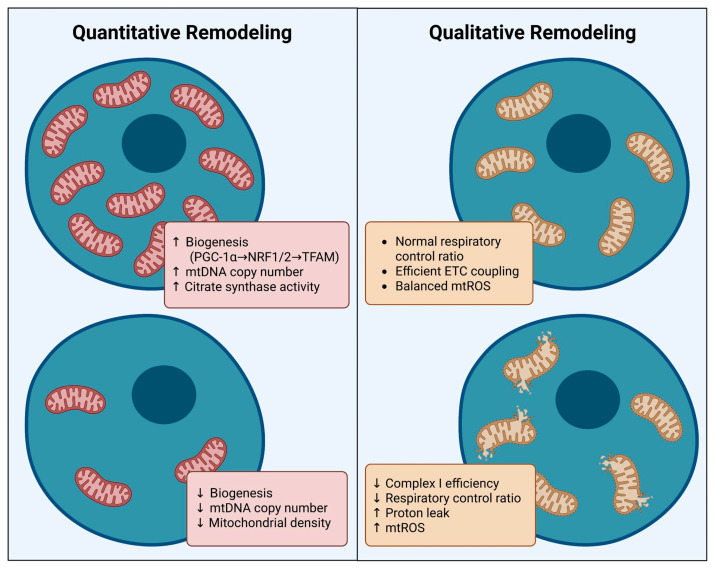
Quantitative versus qualitative mitochondrial remodeling. Mitochondrial adaptations to endocrine and metabolic stimuli can occur through changes in mitochondrial content, referred to as quantitative remodeling, or intrinsic mitochondrial function, referred to as qualitative remodeling [4,5,8,9,57,58,59]. Quantitative remodeling reflects alterations in mitochondrial abundance, including changes in mitochondrial biogenesis, mtDNA copy number, and mitochondrial volume density, often regulated by PGC-1α–NRF1/2–TFAM signaling [13,14,15]. In contrast, qualitative remodeling refers to changes in intrinsic mitochondrial efficiency independent of mitochondrial number, including alterations in electron transport chain activity, respiratory control ratio, proton leak, supercomplex assembly, and reactive oxygen species production [41,42,43,48,49,50,51,52,58,59]. Hormonal transitions may differentially influence these processes, resulting in distinct mitochondrial phenotypes that contribute to disease progression. Up arrows indicate increases while down arrows indicate decreases.

Mitochondria also participate directly in innate immune signaling. Release of mitochondrial DNA into the cytosol can activate cGAS–STING signaling, while cardiolipin exposure and oxidized mitochondrial DNA can promote NLRP3 inflammasome activation [53,54,55,56]. These pathways link mitochondrial dysfunction to inflammatory signaling and provide a mechanistic bridge between endocrine regulation, immunometabolism, and chronic disease. Because these signaling outputs are influenced by both mitochondrial quality and endocrine context, they provide an important foundation for understanding sex-dependent mitochondrial remodeling. These distinct forms of mitochondrial remodeling are illustrated in Figure 2.

## 3. Sex Differences in Mitochondrial Morphology, Dynamics, and Quality Control

Mitochondrial function depends on continuous remodeling through fusion, fission, and selective mitochondrial quality control. Fusion promotes exchange of mitochondrial contents, maintenance of mitochondrial membrane potential, and preservation of cristae architecture, whereas fission facilitates mitochondrial distribution, isolation of damaged mitochondrial segments, and mitophagic clearance [60,61]. Key mediators of these processes include mitofusin 1 and 2 (MFN1/2), optic atrophy protein 1 (OPA1), and dynamin-related protein 1 (DRP1). MFN1/2 regulate outer mitochondrial membrane fusion, OPA1 supports inner membrane fusion and cristae structure, and DRP1-mediated fission contributes to mitochondrial fragmentation under stress conditions [41,60,61,62]. Mitophagy, regulated in part through PINK1/Parkin-dependent pathways, removes damaged mitochondria and limits accumulation of dysfunctional organelles that may otherwise promote mtROS generation and inflammatory signaling [63,64].

Sex differences in mitochondrial morphology and quality control have been reported across multiple tissues, although the direction and magnitude of these differences depend on tissue type, age, hormonal status, and metabolic context. In several experimental models, female tissues demonstrate a more fusion-biased mitochondrial network, enhanced mitochondrial quality control, and greater antioxidant buffering capacity compared with males [29,30,31,65]. These features may help preserve respiratory coupling and limit accumulation of damaged mitochondria during metabolic or oxidative stress. In contrast, male tissues in some contexts show increased susceptibility to mitochondrial fragmentation, impaired mitophagic adaptation with aging, and greater oxidative vulnerability [66,67]. However, these patterns should not be interpreted as universal across all tissues, as mitochondrial dynamics are highly responsive to local metabolic demand, endocrine signaling, and disease state.

Sex hormones may contribute to these differences by regulating mitochondrial biogenesis, dynamics, and stress-response pathways. Estrogen signaling has been associated with increased expression of genes involved in mitochondrial biogenesis and antioxidant defense, while also supporting fusion-associated mitochondrial morphology and mitophagic efficiency in select tissues [10,11,12,13,14,15,16,29,30,31]. Testosterone and androgen receptor signaling also influence mitochondrial structure and quality control, particularly in metabolically active tissues such as skeletal muscle and adipose tissue, although these effects appear highly context dependent. Age-related endocrine changes, including estrogen loss during menopause and declining testosterone levels in males, may further alter mitochondrial dynamics and reduce mitochondrial resilience.

**Figure 3:**
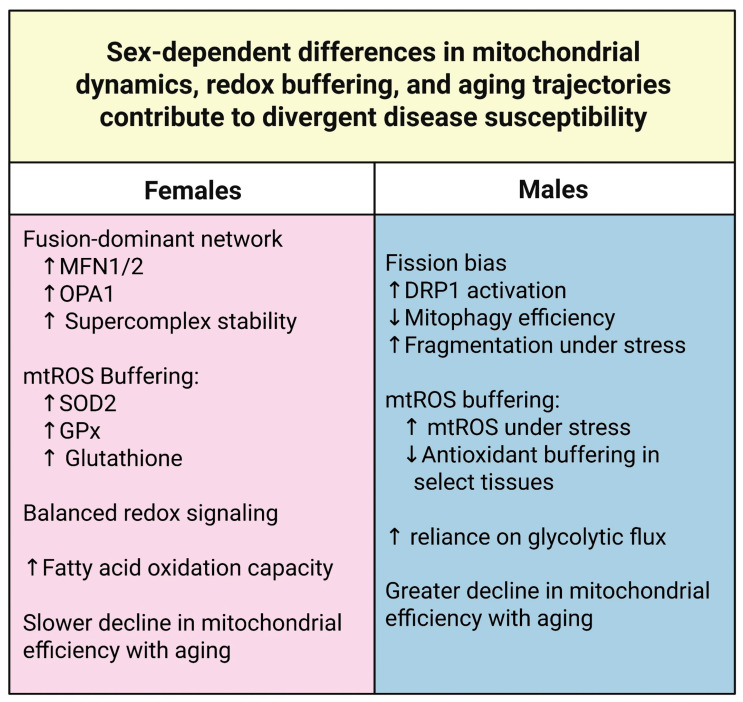
Sex differences in mitochondrial structure, dynamics, and redox regulation. Mitochondrial morphology and function exhibit sex-dependent differences that influence cellular metabolism and stress responses [1,29,30,31,65,66,67]. Female cells are characterized in several experimental contexts by a fusion-biased mitochondrial network, increased expression of fusion proteins, enhanced mitophagy, and greater antioxidant capacity, including greater SOD2 protein abundance and enhanced glutathione-related buffering capacity [29,30,31,60,61,62,63,64,65]. In contrast, male cells in certain contexts exhibit increased mitochondrial fragmentation associated with DRP1-mediated fission, reduced mitophagic efficiency with aging, and increased susceptibility to oxidative stress [29,30,31,62,63,64,65,66,67]. These differences contribute to distinct aging trajectories, with relatively preserved mitochondrial function in females and greater decline in mitochondrial quality in males. Together, these structural and functional differences may influence susceptibility to metabolic and inflammatory diseases. Up arrows indicate increases while down arrows indicate decreases.

Importantly, changes in mitochondrial morphology do not necessarily indicate improved or impaired mitochondrial function without direct bioenergetic assessment. A fused mitochondrial network may support efficient oxidative phosphorylation under some conditions, but excessive fusion can also reflect stress adaptation. Similarly, fragmentation may indicate mitochondrial damage, but it can also facilitate mitophagic clearance and remodeling. Therefore, interpretation of sex differences in mitochondrial dynamics should be paired with functional measurements such as respiratory control ratio, substrate-specific respiration, membrane potential, mtROS production, and mitophagic flux. These structural and functional differences are summarized in Figure 3.

## 4. Sex-Specific Metabolic Pathways and Energy Production

Sex differences in substrate utilization shape mitochondrial bioenergetics and contribute to differences in metabolic flexibility across tissues. In general, females often exhibit greater reliance on fatty acid oxidation during endurance exercise and metabolic stress, whereas males may demonstrate relatively greater carbohydrate utilization in some contexts. However, these differences are influenced by tissue type, age, training status, nutritional state, and circulating sex hormone levels [65,68,69]. Emerging human metabolomic and exercise physiology studies further support enhanced lipid utilization in females, demonstrating sex-specific differences in body composition and circulating metabolite responses to sustained physical training that are consistent with greater reliance on fatty acid oxidation and improved metabolic flexibility in women compared with men [68]. These findings suggest that sex differences in mitochondrial substrate selection are reflected not only at the cellular level, but also in whole-body metabolic adaptation and exercise performance.

Carnitine palmitoyltransferase 1A (CPT1A) serves as a key regulator of mitochondrial long-chain fatty acid transport and β-oxidation, linking hormonal regulation directly to substrate utilization and metabolic disease risk [44,69]. Alterations in CPT1A activity may influence hepatic lipid handling, insulin sensitivity, and inflammatory signaling, highlighting mitochondrial fatty acid transport as an important node connecting sex hormones to systemic metabolic health. Estrogen signaling has been associated with enhanced fatty acid oxidation, AMPK activation, and improved mitochondrial oxidative efficiency in select tissues, although these effects vary across experimental models and endocrine states [10,11,32,33,65,68,69].

Sex-specific differences in oxidative phosphorylation also depend on mitochondrial quality rather than mitochondrial content alone. Estrogen has been associated with improved complex I and IV activity, enhanced respiratory coupling, and increased resistance to oxidative stress in several experimental systems [11,32,33]. However, these outcomes should be interpreted in relation to the assays used, as increased expression of electron transport chain proteins does not necessarily indicate improved respiratory efficiency. Direct measurements of substrate-specific respiration, respiratory control ratio, proton leak, ATP production, and mtROS emission are needed to determine whether observed changes reflect true bioenergetic remodeling.

Sex-specific regulation of redox buffering further influences metabolic and inflammatory tone. Females often demonstrate increased antioxidant enzyme expression, greater SOD2 protein abundance, and enhanced glutathione-related buffering capacity in select tissues, which may limit excessive mtROS accumulation while preserving physiologic redox signaling [29,30,31,70]. These differences may contribute to improved mitochondrial resilience under some conditions, but they may also alter responses to redox-targeted therapies depending on baseline antioxidant capacity and tissue context.

Because immune cell activation is tightly linked to metabolic programming, sex differences in mitochondrial substrate utilization may also influence inflammatory phenotypes. Fatty acid oxidation and oxidative phosphorylation are generally associated with immune tolerance, tissue repair, and regulatory immune states, whereas glycolytic remodeling and excessive mtROS production can promote inflammatory activation [53,54,55,71]. Thus, sex-dependent differences in fuel preference, redox buffering, and mitochondrial respiratory efficiency may contribute to sex-biased immune responses and chronic inflammatory disease vulnerability.

## 5. How Sex Hormones Control Mitochondrial Function

Sex hormones regulate mitochondrial quantity and quality through coordinated genomic, non-genomic, and tissue-specific signaling mechanisms. These effects include transcriptional regulation of mitochondrial biogenesis, modulation of electron transport chain activity, changes in antioxidant defense, and regulation of mitochondrial dynamics and quality control. However, the direction and magnitude of these effects depend on hormone concentration, receptor expression, tissue type, developmental stage, and metabolic state.

Estrogen signaling has been most extensively studied in relation to mitochondrial biogenesis and redox regulation. Estrogen receptors can regulate nuclear transcriptional programs that promote mitochondrial biogenesis through activation of PGC-1α, NRF1/2, and TFAM, which coordinate expression of nuclear and mitochondrial genes required for oxidative phosphorylation [10,11,12,13,14,15]. In select tissues, ERα and ERβ have also been detected in mitochondrial compartments, suggesting that estrogen signaling may influence mitochondrial gene transcription and respiratory function through both nuclear and mitochondrial receptor-associated pathways [10,11,12,16]. These mechanisms support the concept that estrogen can influence both mitochondrial content and intrinsic mitochondrial efficiency.

Estrogen has also been associated with changes in mitochondrial respiratory enzyme activity and antioxidant capacity. Several studies report increased activity of mitochondrial enzymes such as citrate synthase and improved activity of electron transport chain complexes, particularly complexes I and IV, in response to estrogen signaling [11,32,33]. Estrogen signaling has also been linked to increased SOD2 protein abundance and enhanced glutathione-related redox buffering, which may reduce excessive mtROS accumulation while preserving physiologic redox signaling [29,30,31,70]. Importantly, these endpoints represent different levels of mitochondrial regulation: citrate synthase activity reflects enzymatic capacity and mitochondrial content, SOD2 protein abundance reflects antioxidant defense, and respiratory measurements are required to determine whether oxidative phosphorylation capacity is functionally improved.

Androgen receptor signaling also influences mitochondrial metabolism, although its effects are highly tissue- and context-dependent. Testosterone and androgen receptor pathways have been associated with regulation of lipid oxidation genes, insulin signaling, mitochondrial biogenesis, and electron transport chain-related gene expression [17,18,19,20,21]. In skeletal muscle and adipose tissue, androgen signaling may support oxidative metabolism and maintenance of lean mass, while androgen deficiency has been associated with reduced mitochondrial biogenesis, impaired oxidative phosphorylation capacity, increased mtROS production, and altered fatty acid oxidation [22,23,24,25,26,27]. However, androgen excess may produce different effects, particularly in hyperandrogenic states such as PCOS, where altered insulin signaling, inflammation, and mitochondrial stress may converge to impair metabolic flexibility.

Progesterone exerts tissue-specific effects on mitochondrial respiration, calcium handling, and immune adaptation, particularly during pregnancy and reproductive transitions. In addition to classical nuclear progesterone receptor signaling, mitochondrial progesterone receptor pathways have been described in reproductive tissues and may influence mitochondrial membrane potential, oxidative phosphorylation, and ATP production [28]. These findings suggest that progesterone may regulate mitochondrial metabolism through both nuclear transcriptional mechanisms and direct mitochondrial signaling pathways. However, progesterone effects remain less well characterized than estrogen and androgen effects, and additional tissue-specific studies are needed to define its role in mitochondrial function across reproductive and non-reproductive tissues.

Together, these hormone-dependent pathways indicate that sex steroids regulate mitochondria at multiple biological levels, including transcriptional control, protein abundance, enzymatic activity, respiratory function, and quality control. Distinguishing among these levels is essential for interpreting sex hormone effects on mitochondrial biology and avoiding overinterpretation of single endpoints. A summary of hormone-dependent mitochondrial pathways and their disease relevance is provided in Table 1.

## 6. Endocrine Transitions and Pathologic Hormonal States

**Figure 4:**
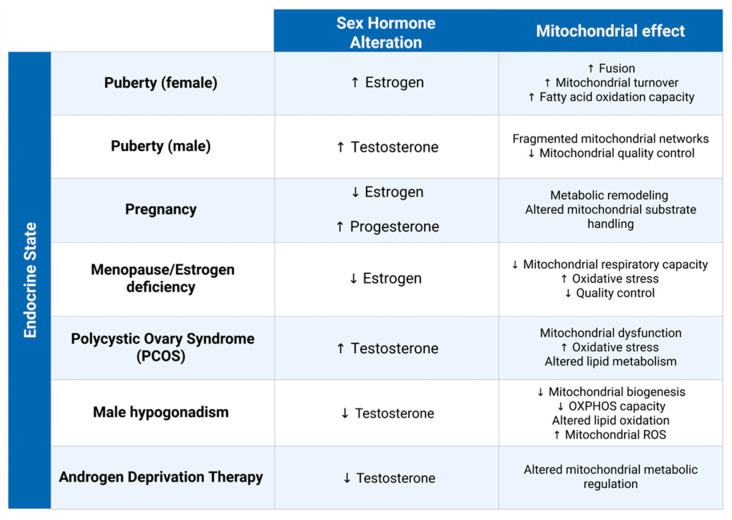
Endocrine states associated with mitochondrial remodeling and disease risk. Physiological and pathological changes in sex hormone levels across the lifespan drive mitochondrial adaptations that may influence disease susceptibility [10,11,12,13,14,15,16,17,18,19,20,21,22,23,24,25,26,27,28,34,35,72,79,80,81,82,83,84,85,86,87,88,89,90,91,92,93,94,95,96]. Puberty, pregnancy, menopause, polycystic ovary syndrome (PCOS), male hypogonadism, and androgen deprivation therapy are associated with distinct hormonal profiles that modulate mitochondrial biogenesis, oxidative phosphorylation, mtROS production, and mitochondrial quality control [17,18,19,20,21,22,23,24,25,26,27,28,34,35,36,37,38,39,40,72,79,80,81,82,83,84,85,86,87,88,89,90,91,92,93,94,95,96]. Estrogen deficiency during menopause is associated with mitochondrial decline and may accelerate age-related bioenergetic dysfunction, while hyperandrogenism in PCOS and androgen deficiency in males may impair mitochondrial metabolism and promote inflammatory signaling [34,35,36,37,38,39,40]. This figure highlights endocrine transitions as key contexts for mitochondrial remodeling and sex-specific disease risk. Up arrows indicate increases while down arrows indicate decreases.

Endocrine transitions and pathologic hormone imbalances provide clinically relevant contexts in which mitochondrial remodeling may influence disease vulnerability. Changes in estrogen, testosterone, and progesterone signaling can alter mitochondrial biogenesis, substrate utilization, redox balance, calcium handling, and quality control. However, mitochondrial responses to hormonal change are highly tissue-specific and depend on age, metabolic status, inflammatory tone, receptor expression, and the functional assays used to evaluate mitochondrial phenotype. Major endocrine contexts associated with mitochondrial remodeling are summarized in Figure 4.

### 6.1. Menopause and Estrogen Deficiency

Estrogen decline during menopause is associated with reduced mitochondrial resilience in several tissues, including vascular endothelium, skeletal muscle, adipose tissue, and brain. Loss of estrogen signaling may impair PGC-1α–NRF1/2–TFAM-mediated mitochondrial biogenesis, reduce antioxidant buffering, alter mitophagy, and increase susceptibility to mtROS accumulation [10,11,12,13,14,15,29,30,31,34]. These changes have been proposed to contribute to increased cardiometabolic and neurodegenerative disease risk after menopause. Importantly, mitochondrial dysfunction may occur before overt clinical disease becomes apparent, suggesting that menopausal transition may represent a window of bioenergetic vulnerability rather than simply a late consequence of aging.

### 6.2. Pregnancy, Postpartum Transition, and Long-Term Mitochondrial Health

Pregnancy represents one of the most profound physiologic examples of endocrine-driven mitochondrial remodeling. Rising estrogen and progesterone levels support coordinated adaptations in mitochondrial biogenesis, oxidative phosphorylation, antioxidant capacity, and immune regulation across maternal tissues [28,72]. These changes are necessary to meet the substantial energetic demands of placental development, fetal growth, vascular adaptation, and maternal metabolic regulation. Mitochondria therefore appear to play an important role in supporting both maternal and fetal health during pregnancy [97,98,99,100,101].

Estrogen promotes transcriptional activation of mitochondrial biogenesis regulators including PGC-1α, NRF1, NRF2, and TFAM signaling, enhances respiratory efficiency, and increases antioxidant defense through transcriptional upregulation of SOD2 and glutathione-related pathways [11,13,31,66]. Progesterone further contributes to tissue-specific mitochondrial regulation through effects on calcium handling, membrane potential, and oxidative phosphorylation, particularly in reproductive tissues and the placenta [28,102]. In addition to classical nuclear progesterone receptor signaling, mitochondrial progesterone receptor pathways may directly regulate ATP production and bioenergetic adaptation during pregnancy, supporting reproductive success and placental function [28].

Placental mitochondria are especially sensitive to hormonal and metabolic disruption [103,104,105]. Abnormal mitochondrial respiration, increased mtROS production, and impaired mitophagy have been implicated in hypertensive disorders of pregnancy, preeclampsia, gestational diabetes, and fetal growth restriction. Excess oxidative stress in placental tissue promotes endothelial dysfunction, inflammatory signaling, and vascular maladaptation, linking mitochondrial dysfunction to both immediate obstetric complications and long-term maternal cardiovascular risk [79,80,103,104,105]. These observations suggest that pregnancy can serve as an early physiologic stress test that reveals underlying mitochondrial vulnerability.

The postpartum period represents a distinct and often underappreciated phase of mitochondrial transition. Following delivery, rapid estrogen withdrawal may reduce mitochondrial biogenesis and antioxidant capacity while increasing susceptibility to oxidative stress. This decline may be particularly relevant in brain regions with high energetic demand, including the hippocampus and prefrontal cortex, where mitochondrial dysfunction can influence mood regulation, cognition, and stress resilience. Reduced bioenergetic reserve during the postpartum period may contribute to vulnerability under physiologic or psychological stress, even when baseline function appears normal [81,82,83].

Emerging evidence suggests that postpartum mitochondrial remodeling may influence long-term neurologic and cardiometabolic health. Women with prior preeclampsia, gestational diabetes, or severe hypertensive disorders of pregnancy exhibit increased risk of later cardiovascular disease, metabolic dysfunction, and cognitive decline [81,82,83]. Persistent mitochondrial dysfunction has been proposed as a mechanistic link between pregnancy complications and later chronic disease risk. Similarly, postpartum changes in mitochondrial signaling may contribute to neuroinflammatory pathways involved in postpartum depression and impaired stress adaptation [81,82,83].

Despite the clinical importance of these transitions, longitudinal studies examining mitochondrial function across pregnancy and postpartum recovery remain limited. Most human studies rely on indirect metabolic markers rather than direct mitochondrial phenotyping, making it difficult to distinguish remodeling from pathologic dysfunction [84]. Future work should integrate high-resolution respirometry, mtROS quantification, mitochondrial DNA damage assessment, and tissue-specific hormone signaling to better define how pregnancy and postpartum transitions shape long-term mitochondrial health.

Recognizing pregnancy and the postpartum period as windows of mitochondrial vulnerability and adaptation may improve risk prediction and therapeutic intervention for women across their lifespan. These stages provide a powerful model for understanding how endocrine transitions reshape mitochondrial function and reveal latent susceptibility to future disease.

### 6.3. Polycystic Ovary Syndrome and Hyperandrogenism

Polycystic ovary syndrome (PCOS) provides a pathologic model of androgen excess, insulin resistance, and chronic low-grade inflammation. Hyperandrogenism and insulin resistance may converge on mitochondrial dysfunction in ovary, skeletal muscle, adipose tissue, immune cells, and the gastrointestinal tract [85,86,87]. Altered mitochondrial respiration, increased mtROS production, impaired substrate utilization, and disrupted antioxidant signaling have been reported in PCOS models and patient-derived samples. However, PCOS is highly heterogeneous, and mitochondrial phenotypes likely differ across lean versus obese PCOS, insulin-resistant versus non-insulin-resistant phenotypes, and varying degrees of hyperandrogenism. Future studies should therefore incorporate endocrine stratification and direct functional mitochondrial measurements to determine whether mitochondrial alterations represent causal contributors, compensatory adaptations, or downstream consequences of metabolic dysfunction.

### 6.4. Male Hypogonadism and Androgen Deprivation Therapy

Male hypogonadism and androgen deprivation therapy also provide clinically relevant models of endocrine disruption and mitochondrial remodeling. Testosterone deficiency has been associated with reduced skeletal muscle oxidative capacity, impaired fatty acid oxidation, increased mtROS production, reduced lean mass, and increased metabolic disease risk [17,18,19,20,21,22,23,24,25,26,27,88]. Androgen deprivation therapy, used commonly in prostate cancer treatment, is associated with increased adiposity, insulin resistance, dyslipidemia, and reduced physical function. These outcomes may involve altered mitochondrial metabolism, although the relative contribution of mitochondrial dysfunction versus broader endocrine and metabolic changes requires further investigation. Distinguishing direct androgen receptor-mediated mitochondrial effects from secondary consequences of altered body composition remains an important area for future research.

### 6.5. Aging, Frailty, and Sarcopenia

Aging is associated with progressive mitochondrial dysfunction that contributes to declining metabolic function, impaired tissue repair, chronic inflammation, and increased susceptibility to cardiometabolic and neurodegenerative disease. Because mitochondrial quality control pathways are strongly influenced by sex hormones, age-related endocrine decline produces sex-specific trajectories of mitochondrial aging and disease vulnerability [1,8,9,24,47,89,90,91,92,93].

In females, the menopausal transition represents a major inflection point in mitochondrial health. Declining estrogen levels reduces mitochondrial biogenesis through impaired PGC-1α–NRF1/2–TFAM signaling, decreases antioxidant defense, and compromises mitophagy efficiency [10,11,13,34,66,94,102]. These changes contribute to increased mtROS production, reduced oxidative phosphorylation capacity, and accumulation of dysfunctional mitochondria in vascular tissue, skeletal muscle, and neural tissues. As a result, postmenopausal women exhibit increased risk of cardiovascular disease, insulin resistance, sarcopenia, and neurodegeneration [29,34,35,95,96,102]. Importantly, mitochondrial dysfunction often precedes overt clinical symptoms, suggesting that mitochondrial decline is not simply a consequence of aging but a driver of disease progression [34].

In males, gradual testosterone decline during aging is associated with reduced mitochondrial oxidative capacity, impaired fatty acid oxidation, and increased skeletal muscle oxidative stress [22,68]. Testosterone deficiency contributes to reduced muscle mass, decreased physical performance, insulin resistance, and increased visceral adiposity, all of which accelerate frailty development [25,26,27,88]. Because androgen receptor signaling regulates mitochondrial metabolism and electron transport chain gene expression, age-related hypogonadism may directly impair mitochondrial function.

Sarcopenia provides a clinically important example of sex-dependent mitochondrial aging. Skeletal muscle requires high mitochondrial turnover to maintain contractile function and metabolic function. Impaired mitophagy, reduced respiratory control ratio, altered mitochondrial dynamics, and increased mtROS all contribute to muscle weakness and loss of lean mass with age [45,46,47,89,92,93]. Female muscle may initially demonstrate greater mitochondrial resilience because of stronger antioxidant buffering and fusion-biased mitochondrial networks, whereas male muscle may show earlier vulnerability to fragmentation and oxidative damage under metabolic stress [30,32,73,93]. However, estrogen withdrawal during menopause may accelerate mitochondrial decline and eliminate this protective advantage.

Frailty is increasingly recognized as a syndrome of impaired bioenergetic reserve in which mitochondrial dysfunction limits physiologic resilience under stress. This concept is particularly relevant to both aging and endocrine disorders, where baseline mitochondrial function may appear preserved, but vulnerability becomes evident during infection, surgery, pregnancy, or metabolic challenge [34,35,74,93]. The distinction between quantitative and qualitative mitochondrial remodeling is especially important in this context, as mitochondrial content may remain relatively stable while respiratory efficiency declines substantially (Figure 2).

These observations support the concept that healthy aging is, in part, a mitochondrial phenotype shaped by endocrine status. Future studies should examine longitudinal mitochondrial changes across menopause, andropause, and frailty progression using tissue-specific mitochondrial phenotyping rather than relying solely on systemic metabolic markers. Better understanding of sex-specific mitochondrial aging may improve early intervention strategies aimed at preserving function and reducing chronic disease burden.

### 6.6. Synthesis: Endocrine Remodeling Requires Tissue-Specific Interpretation

Together, these endocrine states illustrate that mitochondrial remodeling is not a single uniform response to hormone change. Rather, endocrine transitions may alter mitochondrial quantity, intrinsic respiratory quality, redox buffering, substrate utilization, and inflammatory signaling in tissue-specific ways. This framework supports the need for sex-stratified, endocrine-stratified, and tissue-specific mitochondrial phenotyping in studies of aging, reproductive health, cardiometabolic disease, and inflammatory disorders.

## 7. Mitochondria as Hubs Linking Metabolic State to Immune and Stress Responses

Mitochondria integrate nutrient availability with immune activation through mtROS signaling, metabolite flux, and release of mitochondrial danger signals. mtROS can amplify NF-κB signaling and promote NLRP3 inflammasome activation, while mtDNA release activates cGAS–STING pathways [53,54,55,56,71]. These pathways link mitochondrial dysfunction to inflammatory signaling and provide a mechanistic framework for understanding how endocrine state may influence immune activation. Sex differences in antioxidant buffering, substrate utilization, and mitophagy efficiency may therefore shift immune activation thresholds and contribute to sex-biased inflammatory disease vulnerability.

### 7.1. Mitochondria, Gut Barrier Function, and Sex Hormones

The gastrointestinal tract represents a metabolically active and immunologically complex system in which mitochondrial function is essential for epithelial integrity, immune tolerance, and host–microbiome interactions [35]. Intestinal epithelial cells require mitochondrial oxidative phosphorylation to support tight junction assembly, epithelial renewal, nutrient transport, and responses to microbial stress [81]. Disruption of mitochondrial respiration in the gut may increase mtROS, impair epithelial turnover, and contribute to barrier dysfunction, thereby promoting systemic inflammatory and cardiometabolic risk [97].

Sex hormones may influence gastrointestinal mitochondrial function and barrier integrity. Estrogen has been associated with maintenance of epithelial barrier function through effects on mitochondrial biogenesis, antioxidant defense, and tight junction proteins such as occludin, claudins, and zonula occludens-1 (ZO-1) [75,76]. By supporting mitochondrial respiratory efficiency and limiting excessive mtROS accumulation, estrogen may help preserve mucosal homeostasis and reduce susceptibility to inflammatory barrier disruption. These effects may partially explain sex differences in inflammatory bowel disease susceptibility and other immune-mediated gastrointestinal disorders [76,77,78].

**Figure 5:**
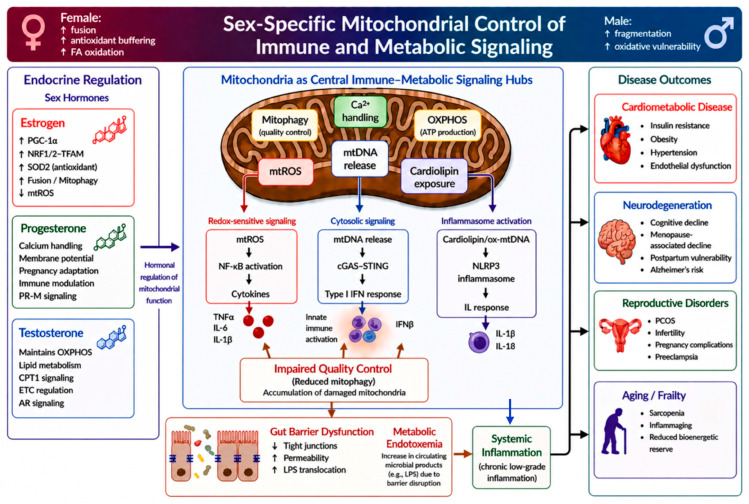
Mitochondria as central immune–metabolic signaling hubs underlying sex-specific disease vulnerability. Sex hormones regulate mitochondrial structure and function through coordinated effects on biogenesis, oxidative phosphorylation (OXPHOS), redox balance, calcium handling, and mitochondrial quality control [10,11,12,13,14,15,16,17,18,19,20,21,22,23,24,25,26,27,28,47,63,64]. Estrogen promotes mitochondrial biogenesis via PGC-1α–NRF1/2–TFAM signaling, enhances antioxidant capacity, and supports fusion-biased mitochondrial networks, whereas testosterone regulates substrate utilization and electron transport chain activity through androgen receptor signaling [10,11,12,13,14,15,16,17,18,19,20,21,22,23,24,25,26,27,29,30,31,32,33,66,67,68,69]. Progesterone exerts tissue-specific effects on mitochondrial metabolism, calcium handling, and immune adaptation, including mitochondrial progesterone receptor-mediated signaling [28,33]. These hormone-dependent effects converge on mitochondria, which function as central signaling hubs integrating metabolic and immune pathways [53,54,55,56,71,106,107,108,109,110,111,112,113,114,115,116,117,118]. mtROS act as redox-sensitive signaling mediators that activate NF-κB and promote pro-inflammatory cytokine production, including TNFα, IL-6, and IL-1β [48,49,50,51,52,106,107,108,109,110,111,112,113]. Release of mitochondrial DNA into the cytosol triggers cytosolic signaling through the cGAS–STING pathway, leading to type I interferon responses and innate immune activation [53,54,56,115,117]. In parallel, cardiolipin exposure and oxidized mtDNA promote NLRP3 inflammasome activation, resulting in interleukin-mediated inflammatory signaling, including IL-1β and IL-18 [42,53,54,55,56,117]. Impaired mitochondrial quality control, characterized by reduced mitophagy and accumulation of damaged mitochondria, may amplify these signaling pathways and contribute to sustained inflammatory responses [63,64,106,107,108,109,110,111,112,113,114,115,116,117,118]. Mitochondrial dysfunction may also disrupt gastrointestinal barrier integrity, leading to increased intestinal permeability, loss of tight junction structure, and translocation of microbial products such as lipopolysaccharide (LPS), which may further promote systemic inflammation [36,37,38,39,75,76,77,78,119,120,121,122]. These interconnected pathways link mitochondrial dysfunction to sex-biased disease outcomes, including cardiometabolic disease, neurodegeneration, reproductive disorders such as PCOS, and aging-associated frailty [1,2,34,35,36,37,38,39,40,123]. Sex-specific differences in mitochondrial dynamics and redox regulation, including enhanced antioxidant buffering and fatty acid oxidation in females and increased susceptibility to oxidative stress and mitochondrial fragmentation in males, may contribute to differential disease vulnerability across the lifespan [29,30,31,65,66,67,68,124,125]. Up arrows indicate increases while down arrows indicate decreases.

In contrast, hyperandrogenic states such as polycystic ovary syndrome (PCOS) may promote colonic mitochondrial dysfunction and contribute to chronic low-grade inflammation. Excess androgen exposure has been associated with impaired mitochondrial respiration, increased mtROS production, epithelial barrier disruption, increased intestinal permeability, and altered microbial composition [36,37,38,39,85,87]. Loss of barrier integrity may facilitate translocation of lipopolysaccharide (LPS) and other microbial products into the systemic circulation, promoting inflammatory signaling through toll-like receptor 4 (TLR4), NF-κB activation, and cytokine production. This gut-derived inflammatory burden may contribute to insulin resistance, vascular dysfunction, and reproductive abnormalities observed in PCOS, although direct causal relationships require further validation as seen in Figure 5 [36].

Mitochondrial dysfunction and gut dysbiosis may reinforce one another in a bidirectional cycle. Increased mtROS can promote inflammatory signaling that alters microbial composition, while dysbiosis may further disrupt mitochondrial metabolism through changes in short-chain fatty acid availability, bile acid signaling, and microbial metabolite production. Reduced butyrate production, for example, may limit colonocyte oxidative metabolism and weaken epithelial barrier maintenance, increasing susceptibility to inflammation [76,119,120]. This interaction places mitochondria at the center of gut–metabolic communication rather than as a secondary consequence of disease alone.

Metformin provides a clinically relevant example of this mitochondrial–gut interaction. Although metformin improves insulin sensitivity and is widely used in PCOS management, it also partially inhibits mitochondrial complex I and alters gut microbiota composition [121,122,126]. In some contexts, these effects may contribute to gastrointestinal symptoms and tissue-specific changes in colonic redox balance despite improving systemic metabolic control [122]. Evaluating metformin through both endocrine and mitochondrial frameworks may therefore provide a more complete understanding of treatment response.

Mitochondria-targeted antioxidants such as MitoTEMPO may offer a potential strategy to interrupt oxidative stress and barrier dysfunction in experimental models. By reducing excessive mtROS, MitoTEMPO may improve epithelial mitochondrial respiration, preserve tight junction integrity, reduce inflammatory cytokine signaling, and support microbiome stability [77,78]. However, the efficacy of such approaches likely depends on baseline redox state, tissue context, biological sex, and endocrine phenotype. Combination strategies using metformin with mitochondrial-targeted antioxidants remain an intriguing possibility, particularly in hyperandrogenic PCOS phenotypes characterized by gastrointestinal inflammation, but require further validation.

Emerging evidence suggests that gut mitochondrial dysfunction may contribute to broader endocrine disease vulnerability beyond PCOS, including obesity and metabolic syndrome [72,123]. Because the intestine functions as both an energetic and immunologic interface with the external environment, mitochondrial dysfunction within this tissue may act as an early amplifier of systemic disease [72]. Future studies should incorporate direct measurement of colonic mitochondrial respiration, intestinal permeability, LPS quantification, and microbiome profiling to better define how sex hormones regulate gut mitochondrial health.

Understanding the mitochondria–gut axis expands the traditional view of mitochondrial biology and highlights a potential therapeutic target for sex-specific metabolic disease. Interventions that improve mitochondrial function may support barrier integrity, reduce inflammation, and improve systemic cardiometabolic outcomes, but these effects should be evaluated using tissue-specific and sex-stratified mitochondrial phenotyping.

### 7.2. Sex Differences in Immunometabolic Programming

Mitochondria function not only as bioenergetic organelles but also as central regulators of immune cell activation, inflammatory signaling, and immunometabolic programming [56,106,107]. Immune responses are tightly linked to metabolic state, and mitochondrial substrate utilization can influence whether immune cells adopt pro-inflammatory, regulatory, or tissue-repair phenotypes. Because sex hormones regulate mitochondrial respiration, mtROS production, and substrate selection, they may also shape sex-specific immune responses and inflammatory disease susceptibility [108].

Macrophage polarization provides a clear example of mitochondria-driven immunometabolism. Classically activated M1 macrophages rely more heavily on glycolysis, exhibit disrupted tricarboxylic acid cycle flux, and generate mtROS that can amplify NF-κB signaling and pro-inflammatory cytokine production. In contrast, alternatively activated M2 macrophages depend more strongly on oxidative phosphorylation and fatty acid oxidation, supporting mitochondrial integrity, tissue repair, and immune tolerance [36,37,38,74,76,77,78,109,110,111,112,113]. Sex hormones may influence this balance, with estrogen generally associated with anti-inflammatory signaling and preservation of mitochondrial respiratory function, whereas testosterone appears to modulate inflammatory tone in a context-dependent manner [108].

T-cell activation is similarly dependent on mitochondrial metabolic programming. Effector T cells require rapid metabolic adaptation and increased glycolytic flux, whereas regulatory T cells and memory T cells rely more heavily on mitochondrial oxidative metabolism and fatty acid oxidation. Mitochondrial calcium handling, NAD+ availability, and respiratory reserve capacity influence T-cell survival and function. mtROS additionally contributes to T-cell activation, differentiation, and effector signaling through redox-sensitive metabolic reprogramming pathways. Estrogen signaling has been shown to alter T-cell activation thresholds and cytokine production, while androgen signaling may suppress excessive inflammatory responses through effects on mitochondrial metabolism and immune cell differentiation [108,114].

mtROS serve as signaling intermediates that coordinate immune activation rather than simply causing oxidative damage. Controlled mtROS production supports redox-sensitive signaling pathways including NF-κB activation, inflammasome priming, and cytokine transcription [121]. However, excess mtROS may contribute to chronic inflammatory signaling, endothelial dysfunction, and tissue injury. Release of mitochondrial DNA into the cytosol activates cGAS–STING signaling and type I interferon responses, while exposure of cardiolipin and oxidized mitochondrial DNA promotes NLRP3 inflammasome activation and IL-1β production (Figure 5) [53,82,115,116,117]. These pathways provide a mechanistic link between mitochondrial dysfunction and innate immune activation.

NADPH oxidases (NOXs) also contribute significantly to cellular redox homeodynamics and may interact with mitochondrial ROS signaling through ROS-induced ROS release mechanisms. Crosstalk between NOX-derived ROS and mitochondrial oxidative signaling can amplify inflammatory responses, disrupt mitochondrial membrane potential, and promote chronic oxidative stress [118]. Emerging evidence suggests sex hormones may differentially regulate NOX expression and activity, although these mechanisms remain incompletely understood and warrant further investigation [127].

Sex differences in these pathways may contribute to the unequal burden of autoimmune and inflammatory diseases between males and females. Females generally demonstrate stronger innate and adaptive immune responses, which may provide protection against infection but also increase susceptibility to autoimmune diseases such as systemic lupus erythematosus, rheumatoid arthritis, and thyroid disease [114]. Enhanced mitochondrial antioxidant buffering and estrogen-mediated immune modulation may preserve immune flexibility under physiologic conditions, but dysregulation of these same pathways may amplify chronic inflammatory disease risk [30].

The interaction between mitochondria and immunometabolism is also highly relevant in aging. Age-related decline in mitophagy and increased accumulation of damaged mitochondria may contribute to a chronic low-grade inflammatory state associated with frailty, neurodegeneration, and cardiometabolic disease. Because menopause and testosterone decline are associated with mitochondrial dysfunction, endocrine aging may contribute to immunometabolic decline [24,34,66,91,128]. This may help explain why hormonal transitions often coincide with increased inflammatory disease burden later in life.

Future therapeutic strategies should recognize that improving mitochondrial function may also influence immune regulation. Mitochondrial-targeted antioxidants, hormone replacement therapy, and therapies that enhance mitophagy or preserve cardiolipin integrity may reduce inflammatory disease burden not only by improving metabolism but also by supporting immunometabolic balance. This framework positions mitochondria as potential regulators of immune tolerance and sex-biased disease vulnerability rather than passive responders to inflammation.

## 8. Clinical Translation and Mitochondrial Therapeutics

Sex-informed mitochondrial biology has important implications for therapeutic development, risk stratification, and precision medicine. Because mitochondrial function is shaped by endocrine state, interventions that target mitochondrial respiration, redox balance, substrate utilization, or quality control may produce sex-specific responses. However, translation of these approaches requires caution because many studies rely on indirect markers of mitochondrial content rather than direct functional measures of bioenergetic capacity. Therapeutic efficacy likely depends on tissue-specific mitochondrial phenotype, baseline redox state, endocrine context, and whether the intervention modifies mitochondrial quantity, intrinsic respiratory efficiency, or both [1,2,12,27,34,40,85].

Mitochondria-targeted antioxidants have received considerable attention as potential therapies for diseases associated with oxidative stress. Conventional antioxidants have often shown limited efficacy in clinical trials, in part because they do not selectively target mtROS and may disrupt physiologic ROS-dependent adaptive responses. Mitochondria-targeted compounds such as MitoTEMPO and MitoQ are designed to accumulate within mitochondria and reduce mitochondrial oxidative stress more directly [129,130]. In experimental models, MitoTEMPO has been associated with improved endothelial function, reduced inflammatory signaling, improved mitochondrial respiratory efficiency, and attenuation of tissue-specific oxidative damage [131]. These effects may be particularly relevant in endocrine and metabolic disorders characterized by increased mtROS, including PCOS, metabolic syndrome, vascular dysfunction, and age-associated inflammatory disease [36,37,38,39,85,86,87].

Importantly, sex differences in mitochondrial redox homeodynamics may substantially modify therapeutic responses to antioxidant-based interventions. Female mitochondria often demonstrate greater antioxidant buffering capacity and enhanced resistance to oxidative injury compared with males in several tissues [29,30,31,124,125]. While this may protect against oxidative damage, it also raises the possibility that excessive antioxidant therapy could suppress physiologic ROS signaling or contribute to reductive mitochondrial stress under certain conditions. Therefore, antioxidant strategies that appear beneficial in males may not produce equivalent effects in females and could potentially impair adaptive mitochondrial signaling if baseline redox buffering is already high. These observations suggest that therapeutic efficacy of mitochondria-targeted antioxidants likely depends on biological sex, tissue context, endocrine status, and pretreatment redox state.

Cardiolipin-stabilizing therapies represent another important area of mitochondrial medicine. Cardiolipin is required for proper electron transport chain organization, cristae architecture, and respiratory supercomplex stability. Disruption of cardiolipin integrity can impair oxidative phosphorylation, promote cytochrome c release, and amplify inflammatory signaling [35,42,77,116,132,133,134]. Elamipretide, a mitochondria-targeted peptide that interacts with cardiolipin and supports inner membrane function, has shown potential benefits in preclinical and early clinical studies involving heart failure, skeletal muscle dysfunction, and mitochondrial disease [116,132,133,134]. Because sex hormones may influence membrane composition, mitochondrial dynamics, and redox buffering, future studies should determine whether cardiolipin-targeted interventions exhibit sex-specific efficacy or tissue-dependent responses.

Metformin provides a clinically relevant example of a therapy with both metabolic and mitochondrial effects. Although metformin is widely used for insulin resistance and PCOS, its mechanisms include partial inhibition of mitochondrial complex I, altered AMP/ATP balance, and activation of AMPK signaling [57,121,122,126,135]. These actions may improve hepatic glucose metabolism and insulin sensitivity, but they can also influence gut microbiota composition, intestinal mitochondrial function, and gastrointestinal tolerance [57,121,122,126,135]. In hyperandrogenic PCOS phenotypes, metformin may improve systemic metabolic parameters while producing variable effects on gastrointestinal symptoms, microbial composition, and tissue-specific mitochondrial redox balance. These observations support the need to evaluate metformin responses using both endocrine and mitochondrial frameworks rather than relying solely on systemic metabolic endpoints.

Exercise remains one of the most effective non-pharmacological interventions for improving mitochondrial function. Exercise stimulates mitochondrial biogenesis through AMPK, SIRT1, and PGC-1α signaling, enhances oxidative phosphorylation capacity, promotes mitophagy, and improves metabolic flexibility [65,68,86,124,125,136]. However, exercise-induced mitochondrial adaptations may differ between males and females because of differences in substrate utilization, hormonal signaling, antioxidant buffering, and respiratory reserve [65,68,124,125]. Females often demonstrate greater reliance on fatty acid oxidation and stronger redox buffering, whereas males may exhibit greater oxidative vulnerability under certain metabolic stress conditions [30,125]. These sex-specific metabolic patterns suggest that exercise prescriptions and mitochondrial outcome measures may require endocrine and sex-specific interpretation.

Hormone-based therapies also have direct mitochondrial relevance. Estrogen replacement has been proposed to support mitochondrial biogenesis, endothelial function, antioxidant defense, and respiratory efficiency, particularly when initiated within an appropriate therapeutic window [10,11,23,30,32,33,40,137]. However, the mitochondrial effects of hormone therapy likely depend on timing, dose, formulation, tissue type, receptor distribution, and baseline cardiovascular or metabolic risk. Similarly, testosterone replacement in hypogonadal males may improve skeletal muscle oxidative metabolism, lean mass, and metabolic function in selected contexts, but broad conclusions require caution because therapeutic responses may vary depending on age, disease status, and cardiovascular risk [23,40]. Thus, hormone-based therapies should be considered potential mitochondrial modulators, but their efficacy and safety require tissue-specific and endocrine-stratified evaluation.

Emerging mitochondrial interventions, including NAD+ restoration, mitophagy enhancement, and modulation of mitochondrial dynamics, may further expand the therapeutic landscape. NAD+ precursors may support mitochondrial metabolism through sirtuin-dependent pathways, while strategies that improve mitophagy may enhance clearance of damaged mitochondria and reduce inflammatory signaling [63,64,138,139,140]. However, these approaches remain incompletely defined in the context of biological sex and endocrine state. Future studies should determine whether interventions that improve mitochondrial quality control have differential effects across menopause, PCOS, hypogonadism, pregnancy-related complications, and aging-associated frailty.

Together, these observations support a more nuanced therapeutic framework in which mitochondrial-targeted interventions are not assumed to act uniformly across biological sex or endocrine context. Clinical and translational studies should incorporate sex-stratified analyses, endocrine phenotyping, and direct mitochondrial outcome measures, including high-resolution respirometry, respiratory control ratio, substrate-specific flux analysis, mitochondrial membrane potential, mtROS quantification, and cardiolipin profiling [53,58,59,84,117]. Such approaches may help distinguish whether an intervention improves mitochondrial content, intrinsic respiratory efficiency, redox balance, or quality control. Incorporating these measures into future trials may improve therapeutic stratification and reduce the risk of overinterpreting indirect mitochondrial endpoints.

## 9. Knowledge Gaps, Translational Challenges, and Future Directions

Despite increasing recognition that biological sex influences mitochondrial biology, several important knowledge gaps remain. A major limitation across the field is the inconsistent use of mitochondrial endpoints. Many studies rely on indirect markers such as mtDNA copy number, electron transport chain protein abundance, citrate synthase activity, or mitochondrial mass to infer mitochondrial health. Although these markers provide useful information regarding mitochondrial content or enzymatic capacity, they do not necessarily reflect intrinsic respiratory efficiency, substrate-specific oxidative capacity, ATP production, proton leak, or mtROS emission. Future studies should therefore combine markers of mitochondrial abundance with direct functional assays, including high-resolution respirometry, respiratory control ratio analysis, substrate-specific flux measurements, mitochondrial membrane potential assessment, ATP production assays, and mtROS quantification [57,58,59].

Another major challenge is the tissue-specific nature of mitochondrial responses to sex hormones. Estrogen, testosterone, and progesterone may exert distinct mitochondrial effects in vascular tissue, skeletal muscle, adipose tissue, immune cells, brain, reproductive organs, liver, and the gastrointestinal tract. These differences likely reflect variation in hormone receptor expression, local metabolic demand, substrate availability, inflammatory environment, and mitochondrial quality control capacity. As a result, findings from one tissue or model system should not be assumed to apply universally across organ systems. Greater emphasis on tissue-specific mitochondrial phenotyping will be essential for distinguishing adaptive remodeling from pathologic dysfunction [1,10,11,12].

Endocrine stratification is also necessary for improving interpretation of sex-dependent mitochondrial findings. Many studies compare males and females without accounting for menopausal status, menstrual cycle phase, pregnancy or postpartum state, contraceptive use, hormone replacement therapy, androgen status, or androgen deprivation therapy. These variables may substantially influence mitochondrial respiration, redox buffering, substrate utilization, and inflammatory signaling. Similarly, studies of PCOS should account for heterogeneity in hyperandrogenism, insulin resistance, obesity, and inflammatory burden [38,85,86,87]. Without careful endocrine stratification, apparent sex differences may reflect unmeasured hormonal variation rather than stable biological sex effects.

There is also a need to better distinguish mechanistic drivers from compensatory adaptations. For example, increased mitochondrial content may represent a beneficial adaptive response, a compensatory response to impaired respiratory efficiency, or a marker of incomplete mitochondrial turnover. Similarly, increased mtROS can function as a physiologic signaling mechanism or contribute to oxidative damage and inflammatory amplification depending on magnitude, duration, and cellular context [53,108,109,110,112,113,115,117]. Future studies should incorporate longitudinal designs and functional assays capable of determining whether mitochondrial changes precede, accompany, or follow disease development.

Multi-omics approaches may help overcome some of these limitations. Integration of transcriptomics, metabolomics, proteomics, lipidomics, and functional mitochondrial phenotyping can provide a more complete view of sex-specific mitochondrial remodeling than any single endpoint alone. Spatial and single-cell approaches may be particularly valuable for identifying tissue-specific mitochondrial signatures within heterogeneous organs such as the brain, intestine, placenta, liver, and immune system. Integration of transcriptomics, metabolomics, proteomics, lipidomics, and functional mitochondrial phenotyping with artificial intelligence-assisted modeling approaches may improve identification of sex-specific mitochondrial signatures, enhance study standardization, and strengthen precision medicine applications [141,142,143,144,145,146,147].

Future translational studies should incorporate sex-stratified analyses, endocrine phenotyping, and mitochondrial functional endpoints as core design features rather than post hoc analyses. This is especially important for studies evaluating mitochondrial-targeted antioxidants, metformin, exercise interventions, hormone-based therapies, NAD+ precursors, and mitophagy-enhancing strategies. Because therapeutic responses likely depend on baseline mitochondrial phenotype, endocrine state, tissue context, and redox balance, standardized mitochondrial phenotyping may improve interpretation of efficacy and reduce the risk of overgeneralization.

Collectively, current evidence supports the concept that sex-dependent mitochondrial remodeling is driven by coordinated differences in substrate utilization, redox buffering capacity, mitochondrial quality control, inflammatory signaling, and endocrine regulation rather than by isolated descriptive associations alone. Ultimately, future research should move beyond describing sex differences toward defining mechanistic principles that explain why those differences occur and how they influence disease susceptibility. A more rigorous integration of mitochondrial physiology, endocrine biology, immunometabolism, and tissue-specific phenotyping will be essential for advancing sex-informed mitochondrial medicine.

## 10. Conclusions

Biological sex is an important determinant of mitochondrial function, shaping how cells respond to metabolic demand, oxidative stress, immune activation, and aging. Mitochondria function not only as bioenergetic organelles but also as endocrine-responsive signaling platforms that integrate hormonal cues with oxidative phosphorylation, redox balance, calcium homeostasis, innate immune signaling, and mitochondrial quality control. Through coordinated effects on mitochondrial biogenesis, substrate utilization, respiratory efficiency, mtROS production, and mitophagy, estrogens, androgens, and progesterone contribute to distinct mitochondrial phenotypes that may influence sex-specific disease vulnerability.

A central concept emerging from this review is that mitochondrial content and mitochondrial function should not be interpreted interchangeably. Quantitative changes in mitochondrial abundance, such as altered mtDNA copy number, citrate synthase activity, or electron transport chain protein abundance, do not necessarily indicate preserved or impaired oxidative phosphorylation capacity. Functional assessment of mitochondrial respiration, substrate-specific flux, respiratory control ratio, membrane potential, ATP production, and mtROS emission is necessary to distinguish adaptive remodeling from bioenergetic dysfunction. This distinction is particularly important when interpreting mitochondrial changes across endocrine transitions, including menopause, pregnancy, postpartum recovery, PCOS, male hypogonadism, androgen deprivation therapy, and aging.

Mitochondrial dysfunction also provides a mechanistic link between endocrine disruption and immunometabolic disease. Excess mtROS, impaired mitophagy, altered substrate utilization, and release of mitochondrial danger signals may activate inflammatory pathways including NF-κB, cGAS–STING, and NLRP3 inflammasome signaling. These pathways can contribute to gut barrier dysfunction, systemic inflammation, vascular dysfunction, insulin resistance, and tissue-specific degeneration. The gastrointestinal tract highlights how mitochondrial dysfunction within a metabolically active barrier tissue may amplify systemic disease by altering epithelial integrity, host–microbiome interactions, and inflammatory tolerance.

Therapeutically, mitochondria represent promising but complex targets. Exercise, hormone-based therapies, metformin, mitochondria-targeted antioxidants, cardiolipin-stabilizing therapies, NAD+ restoration, and mitophagy-enhancing strategies may influence mitochondrial function and disease phenotypes. However, therapeutic responses are unlikely to be uniform across sex, tissue type, endocrine state, or baseline redox status. Sex-specific differences in redox buffering and mitochondrial quality control may alter therapeutic efficacy and, in some contexts, could influence the balance between reducing oxidative damage and preserving physiologic ROS signaling.

Future research should move beyond descriptive comparisons of males and females toward mechanistic, tissue-specific, and endocrine-stratified mitochondrial phenotyping. Integrating high-resolution respirometry, mtROS quantification, mitochondrial membrane potential assessment, cardiolipin profiling, multi-omics approaches, and AI-assisted modeling may improve identification of sex-specific mitochondrial signatures and enhance standardization across studies. Together, these advances may help clarify how endocrine signaling shapes mitochondrial quality across the lifespan and support more precise approaches to disease prediction, prevention, and treatment.

Overall, mitochondria provide a unifying biological framework connecting sex hormones, metabolism, inflammation, aging, and chronic disease. Incorporating sex-specific mitochondrial biology into experimental design and translational research will be essential for improving mechanistic understanding and advancing more individualized approaches to health and disease.

## Figures and Tables

**Table 1:** Sex Hormones, Mitochondrial Pathways, and Disease Relevance. Summary of the effects of estrogen, testosterone, and progesterone on mitochondrial biogenesis, oxidative phosphorylation, reactive oxygen species production, and mitochondrial quality control, along with associated mechanisms and disease implications [10,11,12,13,14,15,16,17,18,19,20,21,22,23,24,25,26,27,28,29,30,31,32,33,66,67,68,69]. Hormonal states including sufficiency, deficiency, and excess are linked to distinct mitochondrial phenotypes that may contribute to cardiometabolic, neurodegenerative, reproductive, and inflammatory disease risk [17,18,19,20,21,22,23,24,25,26,27,28,34,35,36,37,38,39,40,72,73,74,75,76,77,78]. Up arrows indicate increases while down arrows indicate decreases.

Hormonal State	Mitochondrial Effect	Mechanism	Disease Relevance
**Estrogen sufficiency**	↑ Biogenesis, ↓ mtROS	PGC-1α, ERα/β signaling	Cardioprotection
**Estrogen deficiency**	↓ OXPHOS, ↑ inflammation	Impaired mitophagy	CVD, neurodegeneration
**Testosterone sufficiency**	Maintains OXPHOS	Androgen receptor signaling	Metabolic health
**Low testosterone**	↓ Biogenesis, ↑ mtROS	Reduced ETC gene expression	Sarcopenia, T2DM
**Hyperandrogenism**	Mitochondrial stress	Insulin signaling disruption	Metabolic syndrome
**Progesterone**	Tissue-specific effects	Immune/metabolic modulation	Pregnancy adaptations

## Data Availability

No new data were created or analyzed in this study. Data sharing is not applicable to this article.
